# Technology Solutions for Everyday Barriers Among Deaf Older Adults

**DOI:** 10.1093/geroni/igab046.1641

**Published:** 2021-12-17

**Authors:** Megan Bayles, Lyndsie Koon, Shraddha Shende, Wendy Rogers, Jenny Singleton

**Affiliations:** 1 University of Illinois Urbana-Champaign, Champaign, Illinois, United States; 2 University of Kansas, Lawrence, Kansas, United States; 3 University of Texas at Austin, Austin, Texas, United States

## Abstract

American Sign Language (ASL) is the primary form of communication for approximately 250,000 people in the U.S. (Mitchell et al., 2006). As these individuals age, they may experience challenges in their everyday activities. For example, ASL users rely on visual cues, but have age-related change in vision. Moreover, ASL users may need to utilize technology to communicate with non-ASL users, but the technology may not be suitable/usable for older adults. We explored these issues in the Aging Concerns, Challenges, and Everyday Solution Strategies (ACCESS) study, wherein we interviewed Deaf older adults (N=60) in ASL, who provided insights into unique, everyday challenges they encounter. We will focus on the technology solution strategies they incorporate to address and overcome challenges with daily activities. Understanding how participants think about, adapt, and utilize different technologies can inform future technology design to successfully support diverse, aging populations.

